# Brain Health exAmination and Community Knowledge Service: A Primary Care Integrated Neuropsychology Service to Promote Health and Knowledge in Northcentral Florida

**DOI:** 10.1002/alz70860_107685

**Published:** 2025-12-23

**Authors:** Franchesca Arias, Lauren G Santos, Stella Garriga, Agustina Longoni, Laszlo Dimitrakakis

**Affiliations:** ^1^ University of Florida, Gainesville, FL, USA

## Abstract

**Background:**

Residents of rural and partially rural areas are at elevated risk for Alzheimer's disease and related dementias (ADRD) and may have life expectancies reduced by up to 30% for women and 13% for men compared to their counterparts residing in metropolitan areas. Alachua County, the 24th most populous county in Florida, has over 12% of its population aged 65 or older, with about 20% living in poverty. Despite housing several medical institutions, Alachua County faces a shortage of mental health providers and is designated as a Health Professional Shortage Area (HPSA). The Brain Health exAmination and Community Knowledge Service (Brain‐HACKS) aims to promote cognitive and general wellness and promote access to wholistic health services to patients within primary care clinics. This study aims to demonstrate the feasibility for a free community clinic to address the healthcare needs of under‐resourced persons in Alachua County.

**Method:**

Persons learn about the service through flyers, word of mouth, or via referrals. Up to seven 30‐minute appointments are scheduled quarterly on Saturdays from 8am to 12pm. Appointments include a clinical interview and a reading assessment as well as measures of cognition and social factors. Patients receive feedback and information about community resources. Phase 1 of Brain‐HACKS extended from January 2024 to October 2024, and involved selection of instruments, collaboration with partners, development of resources, and recruitment of trainees. Phase 2 began in November 2024 and involves provision of services and refinement of the service processes.

**Result:**

Eight patients have been evaluated. Overall, patients were female (57%), underinsured (80%), unemployed or under‐employed (74%), and from Non‐Hispanic Black or Latina(o) descent (70%). Interest among emerging professionals was documented. We recruited 2 graduate trainees and 2 undergraduate trainees who are supervised by a licensed clinical neuropsychologist.

**Conclusion:**

Preliminary findings suggest that Brain‐HACKS is being well received among community members and provides services to persons with limited access to cognitive and mental health providers. Brain‐HACKS is an innovative way to enhance public health and to potentially repair mistrusts with community members. These findings offer critical insight into resource allocation and partnership strategies needed to develop and sustain a service.

